# A Smoking Gun? Epigenetic Markers of Tobacco Use History

**DOI:** 10.1289/ehp.122-A56

**Published:** 2014-02-01

**Authors:** Julia R. Barrett

**Affiliations:** Julia R. Barrett, MS, ELS, a Madison, WI–based science writer and editor, has written for *EHP* since 1996. She is a member of the National Association of Science Writers and the Board of Editors in the Life Sciences.

Epigenetic changes are associated with disease processes as well as with specific environmental exposures related to toxic agents, nutritional factors, and lifestyle.^1^^,^^2^ Our unfolding knowledge of epigenetics may provide the framework for better understanding disease processes and how they are modified by environmental factors; eventually investigators may identify biomarkers for early disease detection and treatment.^2^ A new study in *EHP* now shows that past exposures can be detected and possibly even quantified based on the epigenetic footprints they leave on specific genes.^3^

The study centered on smoking and methylation of the *F2RL3* gene, which is involved in platelet activation and, potentially, certain cardiovascular functions.^3^^,^^4^ Epigenetic changes are represented by molecular tags tacked onto genes that affect their expression; in methylation, these tags are methyl groups. Environmental exposures and their timing can influence the degree of DNA methylation and the related consequences.^5^^,^^6^

In a previous study, several members of the research group found that the *F2RL3* gene was hypomethylated (or less intensely methylated) in smokers than in nonsmokers.^4^ In a subsequent study, they identified an association between *F2RL3* hypomethylation and increased mortality among individuals with stable coronary heart disease.^7^

The current study included data for 3,588 participants aged 50–75, which was collected through ESTHER, a cohort study of older adults in southwest Germany.^8^ Upon enrollment in 2000–2001, study participants completed a detailed questionnaire covering sociodemographic and lifestyle characteristics, medical history, and tobacco use. Medical records and self-report were used to identify individuals with disorders such as diabetes, hypertension, and cardiovascular disease. The participants also provided blood samples that were analyzed to pinpoint methylation patterns.

**Figure:**
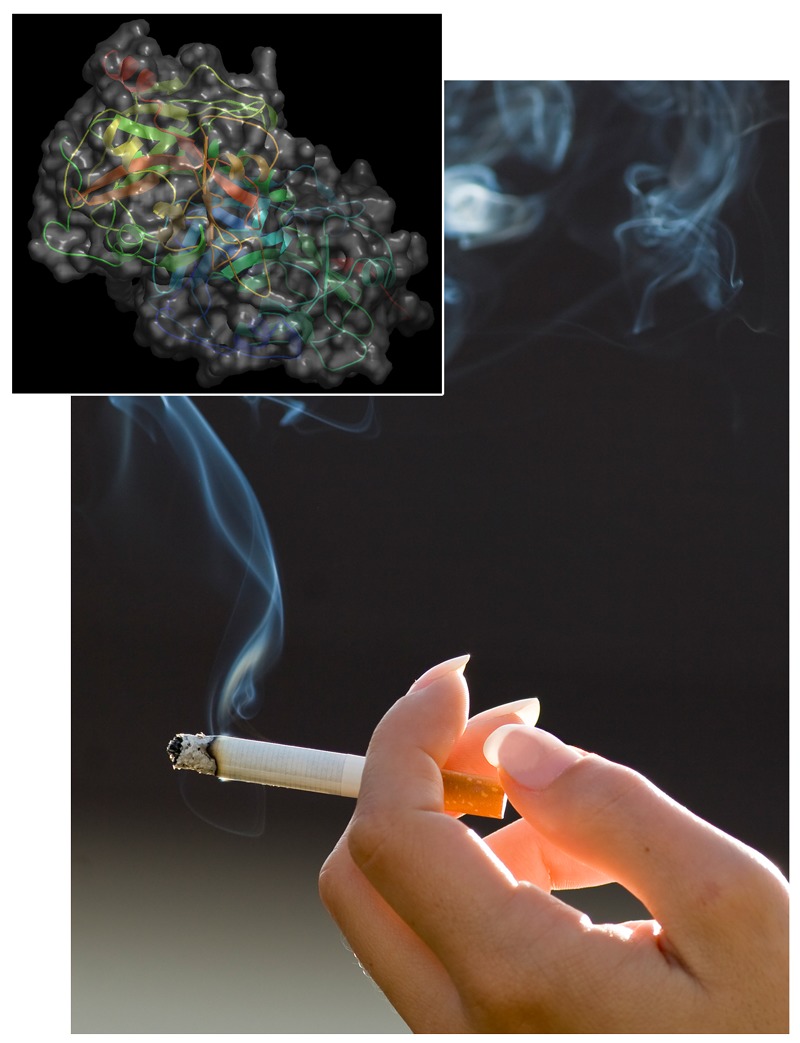
Methylation of the *F2RL3* gene (inset) could offer a highly accurate measure of smoking history. Smoke: © maksdezman/iStock; gene structure: © Mike Hartshorn

The investigators zeroed in on smoking behaviors, comparing 1,136 former smokers, 654 current smokers, and 1,701 never-smokers. For smokers, the researchers examined *F2RL3* methylation in relation to age at smoking initiation, years smoked (duration), cumulative pack-years (dose), and, as applicable, how much they currently smoked (intensity) or how long since they had quit.

All analyses were highly consistent not only in indicating the link between *F2RL3* hypomethylation and smoking, but most importantly in identifying a highly consistent dose–response relationship with cumulative smoking. In particular, current smoking intensity and duration of smoking were inversely related to the degree of methylation. Young age at initiation also was associated with less intense methylation. Further, the researchers found that methylation increased with elapsed time since quitting, returning to nonsmoking levels at 20–25 years.^3^

“Our new study was much larger in size than the initial study and covered a much broader range of smoking exposure. This enabled a much more detailed analysis of the association not only for current smoking but also for past smoking,” says coauthor Hermann Brenner, a professor at the German Cancer Research Center in Heidelberg.

Although a case can be made for linking the *F2RL3* gene with smoking-related diseases, the researchers caution that the clinical relevance of their findings requires further research. Furthermore, methylation was analyzed at only one point in time, which precluded analyzing changes over time. Unmeasured factors also may have influenced the results.

Nevertheless, the study represents an important advance in epidemiological research, says Andrea Baccarelli, an associate professor of environmental epigenetics at the Harvard School of Public Health, who was not involved in the study. “The finding that *F2RL3* hypomethylation is associated with both duration and intensity of exposure is highly significant,” he says.

Current methods of measuring exposure rely on self-report and biomarker levels in biological samples. The former can be biased, and the latter do not reflect the duration of smoking, much less the long-term history as reflected in cumulative dose, which Baccarelli says is the most relevant factor in predicting diseases caused by smoking. “In *F2RL3* methylation, we potentially have a biomarker that can detect how much and how long people have smoked during their entire lifetime,” he says.

On a broader scale, this approach may be applicable to other genes and possibly other types of exposures. “This study is important because it may create a paradigm—what we are exposed to leaves a record in our epigenome, and this record may be accessed through a simple DNA methylation analysis,” Baccarelli says. This information would be especially helpful for characterizing exposures for which quantification is even more elusive than it is for smoking.

First, however, the current findings need to be confirmed. This work is already under way, says Brenner, along with efforts to investigate the association of *F2RL3* methylation with various disease end points and to identify other genes that undergo epigenetic modification due to smoking.
